# Special pathogen infections presenting with neck mass as the initial manifestation

**DOI:** 10.3389/fcimb.2026.1767591

**Published:** 2026-05-08

**Authors:** Jinnian He, Yan Ning, Hanlin Liang, Jiaqi Qin, Yan Wei, Siqiao Liang, Zhiyi He, Shihua Yin

**Affiliations:** 1 Department of Otolaryngology-Head and Neck Surgery, The Second Affiliated Hospital of Guangxi Medical University, Nanning, Guangxi, China; 2 Department of Respiratory and Critical Care Medicine, The First Affiliated Hospital of Guangxi Medical University, Nanning, Guangxi, China; 3 Wuming Hospital of Guangxi Medical University, Nanning, Guangxi, China; 4 Department of Pathology, The First Affiliated Hospital of Guangxi Medical University, Nanning, Guangxi, China

**Keywords:** immunodeficiency, infection, neck mass, nontuberculous mycobacteria, *Talaromyces marneffei*

## Abstract

**Background:**

The etiology of neck masses is complex. Infections caused by *Talaromyces marneffei* (TM) and *nontuberculous mycobacteria* (NTM) are uncommon but often present with insidious clinical manifestations, leading to frequent misdiagnosis.

**Methods:**

We collected and analyzed data from 13 patients with TM/NTM infections presenting with neck masses at The First Affiliated Hospital of Guangxi Medical University and The Second Affiliated Hospital of Guangxi Medical University. Clinical manifestations, laboratory findings, infection sites, pathogen types, treatments, and outcomes were described and analyzed.

**Results:**

Of the 13 patients, six were male and seven female, with a median age of 57 years (range, 27–73 years). All patients were residents of Guangxi and tested positive for anti-interferon-γ autoantibodies (AIGAs), with titers of 1:2500 in 12 patients and 1:500 in one. The median time from symptom onset to diagnosis was 5 months (range, 1–19 months). Common clinical features included lymphadenopathy (13/13), fever (11/13), respiratory symptoms (10/13), and rash or skin ulceration (8/13). Frequent laboratory abnormalities included leukocytosis (11/13), neutrophilia (11/13), elevated erythrocyte sedimentation rate (12/13), and elevated C-reactive protein (13/13). Coinfection with two or more pathogens was observed in 12 patients. The lungs and lymph nodes were involved in all 13 patients, followed by bone (11/13), skin or soft tissue (8/13), bloodstream or bone marrow (3/13), and nasopharynx (3/13). Neck mass specimens yielded NTM in nine cases and TM in four. NTM was most frequently identified by metagenomic next-generation sequencing (mNGS), whereas TM was detected by culture. The median follow-up duration was 28 months (range, 1–86 months). During follow-up, 6 patients (46.2%) experienced disease exacerbations. Among the 13 patients, 12 achieved clinical improvement after pathogen-directed antimicrobial therapy, while one patient died.

**Conclusion:**

Neck masses have diverse etiologies. TM and NTM infections presenting initially as neck masses are rare and easily misdiagnosed as tuberculosis, malignancy, or lymphoma. Culture and mNGS are crucial diagnostic tools for TM and NTM, respectively. Clinicians should maintain a high index of suspicion for these infections, particularly in immunocompromised patients in endemic regions.

## Introduction

*Talaromyces marneffei* (TM), formerly known as *Penicillium marneffei*, was first isolated from bamboo rats in Vietnam in 1956 (Cao et al., 2019; Morris et al., 2024). This fungus is the only temperature-dependent dimorphic fungus in the genus *Talaromyces*, growing in a filamentous form at 25 °C and converting to a yeast form at 37 °C (Klein and Tebbets, 2007). *Talaromyces marneffei* is predominantly endemic in Southern China and Southeast Asia. Patients with acquired immunodeficiency syndrome (AIDS), malignant tumors, organ transplants, and autoimmune diseases constitute its primary susceptible population (Limper et al., 2017; Morris et al., 2024). Neck masses represent a common clinical presentation with a diverse and complex etiology, encompassing infectious, neoplastic, autoimmune, and congenital disorders. Among infectious causes, beyond typical pyogenic bacterial infections and *Mycobacterium tuberculosis* (TB) infections, certain uncommon pathogens such as TM and NTM can also cause lymph node or soft tissue lesions in the neck (Sun et al., 2020). However, due to their atypical clinical manifestations and the limited sensitivity of conventional diagnostic methods, these infections often lead to misdiagnosis or delayed diagnosis.

Patients with TM infection may present with disseminated disease affecting the skin, lymph nodes, lungs, liver, and spleen. Cervical lymphadenopathy may be the initial or predominant manifestation (Supparatpinyo et al., 1994; Vanittanakom et al., 2006; Chan et al., 2016). Diagnosis is challenging because symptoms are nonspecific and the organism’s histopathologic morphology closely resembles that of *Histoplasma*, *Cryptococcus*, and other pathogens (Chan et al., 2016; Chen et al., 2017). Lymph nodes are common targets of NTM, and NTM lymphadenitis—typically unilateral, painless, and cervical—is relatively common in children (Wolinsky, 1995; Lindeboom, 2012; Liang et al., 2025). It is frequently misdiagnosed as tuberculous or bacterial lymphadenitis. Because NTM is poorly susceptible to conventional antituberculosis drugs, delayed or inaccurate diagnosis prolongs the disease course and may lead to chronic sinus tract formation or the need for surgery (Lindeboom, 2012).

Clinical awareness of TM and NTM infections presenting as neck masses remains low, particularly in non-endemic regions or immunocompetent patients, leading to frequent misdiagnosis. Enhancing understanding of their clinical spectrum, diagnostic workup, and therapeutic strategies is therefore critical for timely treatment and improved prognosis. This article systematically details the epidemiology, clinical manifestations, diagnosis, and treatment principles to serve as a clinical reference.

## Materials and methods

### Study population

We retrospectively analyzed cases of special pathogenic infections (including TM and NTM) presenting with neck masses as the initial symptom, admitted to the First Affiliated Hospital of Guangxi Medical University and The Second Affiliated Hospital of Guangxi Medical University between January 2017 and September 2025.

#### Inclusion criteria

(1) Patients with confirmed TM or NTM infection based on positive culture of clinical specimens (e.g., blood, secretions, bone marrow, lymph nodes, or lung tissue) or positive mNGS; (2) Patients presenting with neck mass as the initial symptom; (3) Complete clinical data available for review.

#### Exclusion criteria

(1) Patients with neck masses caused by other pathogens (e.g., bacteria, viruses, or other fungi); (2) Patients with missing critical clinical data; (3) Patients with concurrent malignancies or other conditions that might confound the diagnosis of neck masses.

### Definition of clinical outcomes

Clinical improvement was defined as resolution or significant improvement of symptoms and signs, with sustained normalization or marked reduction of inflammatory markers (CRP, ESR, PCT), without the need for hospitalization. Disease worsening was defined as clinical deterioration with recurrence or persistence of symptoms and signs, or worsening of laboratory findings after initial improvement, ultimately requiring hospitalization. This included AIGAs-positive patients with recurrent NTM, TM, or other opportunistic infections. Patients who initially stabilized but later worsened due to incomplete treatment were also considered to have disease worsening. Death was considered an endpoint event.

Data collected included demographic characteristics, clinical features, imaging findings, laboratory test results, histopathological findings, and clinical outcomes. In compliance with the strict regulations of the Ethics Committee of the First Affiliated Hospital of Guangxi Medical University for retrospective studies, written informed consent (signed by the patients or their immediate family members) was obtained from all participants prior to the study.

### Methods used to diagnose pathogen infections

NTM Infection: Diagnosis followed the “Guidelines for the Diagnosis and Treatment of NTM Disease” (Daley et al., 2020).

TM Infection: Diagnosis was confirmed by any of the following criteria: (a) detection of TM-specific sequences in qualified clinical samples (e.g., bronchoalveolar lavage fluid, lymph node aspirate, purulent exudate, blood, or sputum) by mNGS; (b) preliminary identification based on colony morphology and rose-red pigment production after incubation on Sabouraud’s agar at 25 °C or 37 °C for 2–3 weeks, with final confirmation by matrix-assisted laser desorption/ionization time-of-flight mass spectrometry (MALDI-TOF MS); or (c) histopathological identification of characteristic TM fungal spores.

Other Pathogens: NGS, microbial culture, and histopathological examination were also used to diagnose infections caused by other pathogens, including viruses, bacteria and various fungi.

In this study, mNGS testing was conducted by either Nanjing Difei Medical Laboratory Co., Ltd. or Tianjin Genskey Medical Laboratory Co., Ltd.

### Measurement of AIGAs titer and neutralizing capacity

Indirect enzyme-linked immunosorbent assay (ELISA) was used to determine the titer and positivity of AIGAs. Serum was isolated from 5 mL peripheral venous blood. Recombinant human IFN-γ (Sino Biological, catalog no. 11725-HNAS) was coated as antigen, and sera were diluted 1:100 with 5-fold serial dilutions to 1:500 and 1:2500. Samples with OD450 > 0.5 and >2-fold of healthy controls were defined as positive.

The neutralizing capacity of AIGAs was evaluated by western blot. PMA-differentiated THP-1 macrophages were incubated with serum-IFN-γ mixtures. Low or absent p-STAT1 indicated positive neutralizing capacity, and normal p-STAT1 indicated no neutralizing activity, which was quantified by ImageJ.

### Statistical analysis

Statistical analyses were performed using SPSS version 26.0. Continuous variables were expressed as mean ± standard deviation (SD) for normally distributed data and as median with interquartile range (IQR) for non-normally distributed data. Categorical variables were presented as frequencies (percentages). Normality was assessed using the Shapiro-Wilk test, and homogeneity of variances was evaluated using Levene’s test. For comparisons between two groups, the independent samples t-test was used for normally distributed data with equal variances, whereas the Mann-Whitney U test was applied for non-normally distributed data. Categorical variables were compared using the chi-square (χ²) test, or Fisher’s exact test when expected frequencies were <5. A two-tailed p-value < 0.05 was considered statistically significant.

## Results

### Demographic and clinical characteristics

A total of 13 patients (6 male, 7 female) native to Guangxi were enrolled, with a median age of 57 years (range, 27–73 years). All patients were diagnosed with AIGAs-associated adult-onset immunodeficiency (AOID) (**Table 1**).

**Table 1:** Demographic and clinical characteristics.

ID	Sex/age	Comorbidity	Pathogenic bacteria	Affected site	Diagnostic methods
P1	F/27	AIGAs associated AOID	TM, *M. fortuitum*, *Aspergillus*	Lymph nodes, lungs, skin and soft tissues, bones, blood	NGS, culture, histopathology
P2	F/64	AIGAs associated AOID, Type 2 diabetes mellitus	*M. abscessus, S. haemolyticus*	Lymph nodes, lungs,bones	NGS
P3	F/51	AIGAs associated AOID	TM, *M. avium, M. abscessus*, EBV	Lymph nodes, lungs, skin and soft tissues, bones, parotid gland	NGS, culture, histopathology
P4	F/44	AIGAs associated AOID	*M. gordonae, S. haemolyticus*, HCMV	Lymph nodes, lungs, skin and soft tissues, blood	NGS
P5	M/62	AIGAs associated AOID	*M. intracellulare, S. maltophilia*	Lymph nodes, lungs,bones	NGS
P6	M/57	AIGAs associated AOID, Hepatitis B-related cirrhosi	*M. colombiense, K. pneumoniae*, HCMV, *A. flavus*	Lymph nodes, lungs, bones, nasopharynx	NGS, culture
P7	F/63	AIGAs associated AOID	TM, EBV	Lymph nodes, lungs, skin and soft tissues, bones	NGS, culture
P8	M/42	AIGAs associated AOID	*M. gordonae, A. baumannii*, EBV	Lymph nodes, lungs, skin and soft tissues, bones	NGS, histopathology
P9	M/57	AIGAs associated AOID, Hypertension	TM, *A. baumannii*, EBV	Lymph nodes, lungs, skin and soft tissues, bones	NGS, culture
P10	M/45	AIGAs associated AOID	TM, *S. enterica*, E. coli, *A. terreus*	Lymph nodes, lungs, skin and soft tissues, bones, blood	NGS, culture
P11	F/50	AIGAs associated AOID, Asthma	TM, *M. intracellulare, A. flavus*	Lymph nodes, lungs, bones, blood	Culture
P12	M/67	AIGAs associated AOID	*A. fumigatus, M. fortuitum*, EBV	Lymph nodes, lungs	NGS
P13	F/73	AIGAs associated AOID, Hypertension, Type 2 diabetes mellitus, Heart disease	*M. abscessus*	Lymph nodes, lungs, skin and soft tissues, bones	NGS

AIGAs, anti-IFN-γ autoantibodies; AOID, adult-onset immunodeficiency disease; *M. fortuitum, Mycobacterium fortuitum; M. gordonae, Mycobacterium gordonae; M. intracellulare, Mycobacterium intracellulare; M. colombiense, Mycobacterium colombiense; A. fumigatus, Aspergillus fumigatus; A. flavus, Aspergillus flavus; A. terreus, Aspergillus terreu; S. haemolyticus, Staphylococcus haemolyticus; S. maltophilia, Stenotrophomonas maltophilia; K. pneumonia, Klebsiella pneumoniae; A. baumannii, Acinetobacter baumannii; S. enterica, Salmonella enterica; E. coli, Escherichia coli; HCMV, Human cytomegalovirus.*

**Figure 1:**
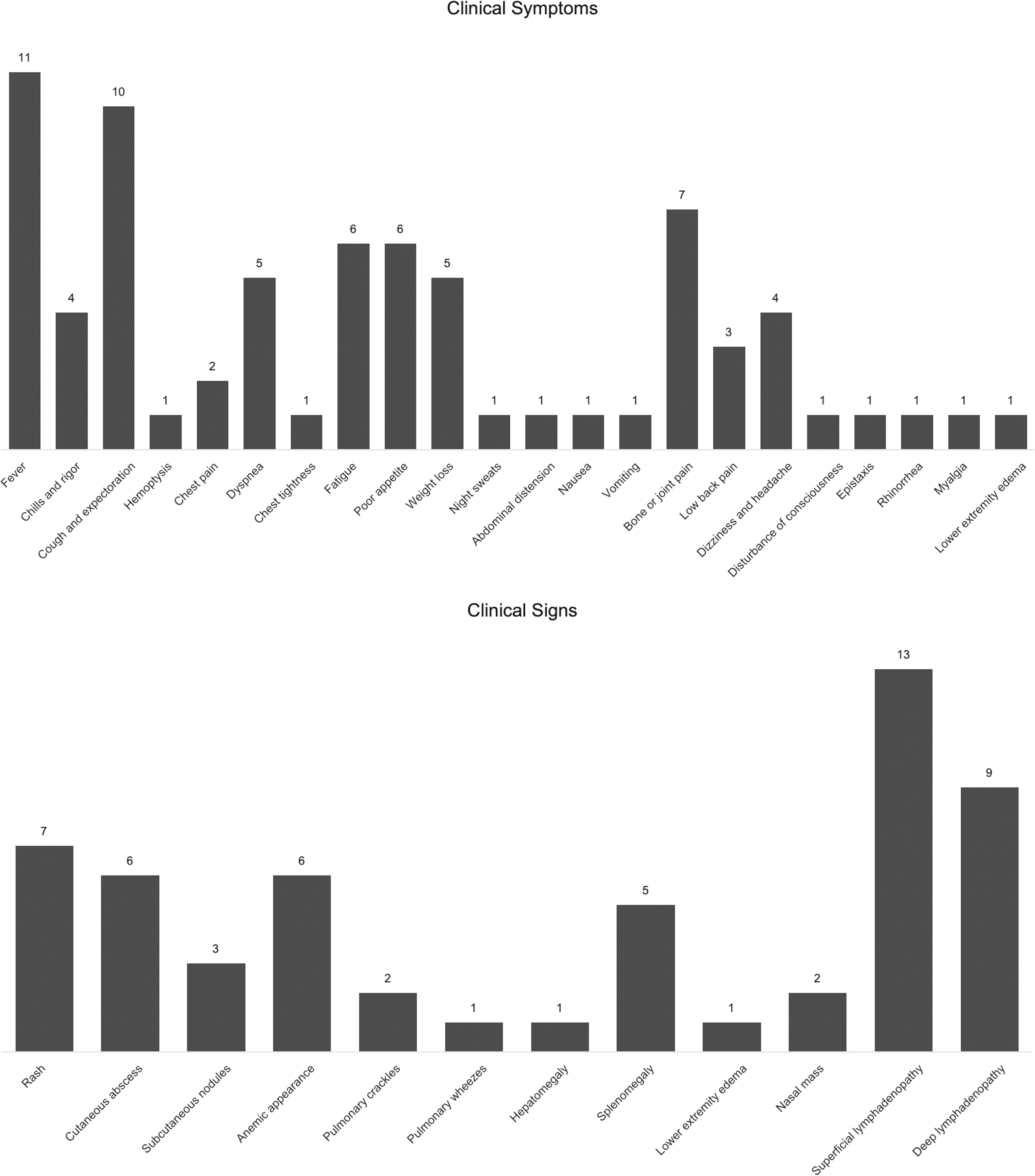
Clinical symptoms and signs of 13 patients with TM/NTM infections presenting with neck masses.

The most common clinical manifestations were lymphadenopathy (13 cases, 100%), fever (11 cases, 84.6%), cough and expectoration (10 cases, 76.9%), rash or skin ulceration (8 cases, 61.5%), and bone or joint pain (7 cases, 53.8%). Other symptoms included dyspnea (5 cases, 38.5%), fatigue (6 cases, 46.2%), poor appetite (5 cases, 38.5%), chills and rigors (4 cases, 30.8%), dizziness and headache (4 cases, 30.8%), low back pain (3 cases, 23.1%), chest pain (2 cases, 15.4%), and hemoptysis, weight loss, night sweats, nausea, vomiting, and lower extremity edema (1 case each, 7.7%) (**Figure 1**).

Comorbidities included hypertension (2 cases), type 2 diabetes mellitus (2 cases), chronic HBV-related cirrhosis (1 case), and cardiac disease (1 case) (**Table 1**).

All patients had multisite infections, most commonly involving the lungs and lymph nodes (13 cases each, 100%), followed by bone (11 cases, 84.6%), skin and soft tissue (8 cases, 61.5%), bloodstream or bone marrow (3 cases, 23.1%), and nasopharynx (3 cases, 23.1%) (**Table 1**).

Pathogens identified included NTM in 10 cases (76.9%), TM in 6 cases (46.2%), EBV in 5 cases (38.5%), *Aspergillus species* in 4 cases (30.8%), *Acinetobacter baumannii* in 2 cases (15.4%), and *cytomegalovirus* (CMV) in 2 cases (15.4%). Coinfection with two or more pathogens was observed in 12 patients (92.3%) (**Table 1**).

### Laboratory findings

A total of 13 patients with TM/NTM infections presenting with neck masses were analyzed. Hematological abnormalities included anemia (mean Hb 87.63 ± 20.82 g/L), leukocytosis in 11 patients (84.6%) with a mean WBC of 15.21 ± 4.38×10^9^ L, and neutrophilia in 11 patients (84.6%) with a mean neutrophil count of 11.22 ± 3.49×10^9^ L. Inflammatory markers were elevated in the majority of patients. All 13 patients (100%) had elevated CRP, with a median of 67.91 [59.16–158.56] mg/dL; 12 patients (92.3%) had elevated ESR, with a mean of 85.15 ± 35.92 mm/h; and 12 patients (92.3%) had elevated PCT, with a median of 0.29 [0.06–0.50] ng/mL. Immunologically, hypergammaglobulinemia was observed, with a mean IgG level of 26.85 ± 9.56 g/L. Median IgE was 112.55 [67.33–223.20] IU/mL, and median IgG4 was 1.07 [0.52–3.16] g/L. Lymphocyte subset analysis revealed preserved T cell subset proportions (CD4 ++ 30.78 ± 9.66%, CD8 ++ 30.86 ± 10.68%). All tested positive for AIGAs, with 12 cases showing a titer of 1:2500 and one case showing a titer of 1:500 (**Table 2**).

**Table 2:** Laboratory data of 13 patients with TM/NTM infections presenting with neck masses.

Variables	Mean±SD/IQR	Reference range
Hb, g/L	87.63±20.82	115-150
WBC, x10^9^/L	15.21±4.38	3.5-9.5
Neu, x10^9^/L	11.22±3.49	1.5-6.3
Lym, x10^9^/L	1.83±0.42	1.1-3.2
Eos, x10^9^/L	0.50[0.31-1.78]	0.02-0.52
Platelet, x10^9^/L	423.28±91.38	125-350
Total T cell count, %	66.69±11.45	30-46
CD4+T cells, %	30.78±9.66	19.2-33.6
CD8+T cells, %	30.86±10.68	62.6-76.8
NK cells, %	21.78±11.59	9.5-23.5
B cells, %	8.67±4.78	8.5-14.5
IgG(g/L)	26.85±9.56	8.6-17.4
IgM(g/L)	1.18±0.66	0.5-2.8
IgA(g/L)	2.41±1.02	1.0-4.2
IgE(IU/ml)	112.55[67.33-223.20]	<100
IgG4(g/L)	1.07[0.52-3.16]	0-2
GLB(g/L)	47.56±12.38	20-40
ALB(g/L)	31.08±4.64	40-55
CRP(mg/dl)	67.91[59.16-158.56]	<10
ESR(mm/h)	85.15±35.92	0-20
PCT(ng/ml)	0.29[0.06-0.50]	<0.05
ALT(U/L)	13.00[9.50-21.00]	7-40
AST(U/L)	13.00[13.00-20.00]	13-35
TBIL(μmol/L)	6.93±3.52	0-21
CHE(U/L)	5145.77±2214.30	5000-12000
Cr(μmol/L)	83.58±40.34	41-73
FIB(g/L)	5.33[4.69-5.51]	2-5
D-dimer, mg/L	569.00[331.00-877.00]	0-450

Reference ranges were established by the Clinical Laboratory of the First Affiliated Hospital of Guangxi Medical University (ISO15189 certified).

### Cervical presentation

**Figure 2:**
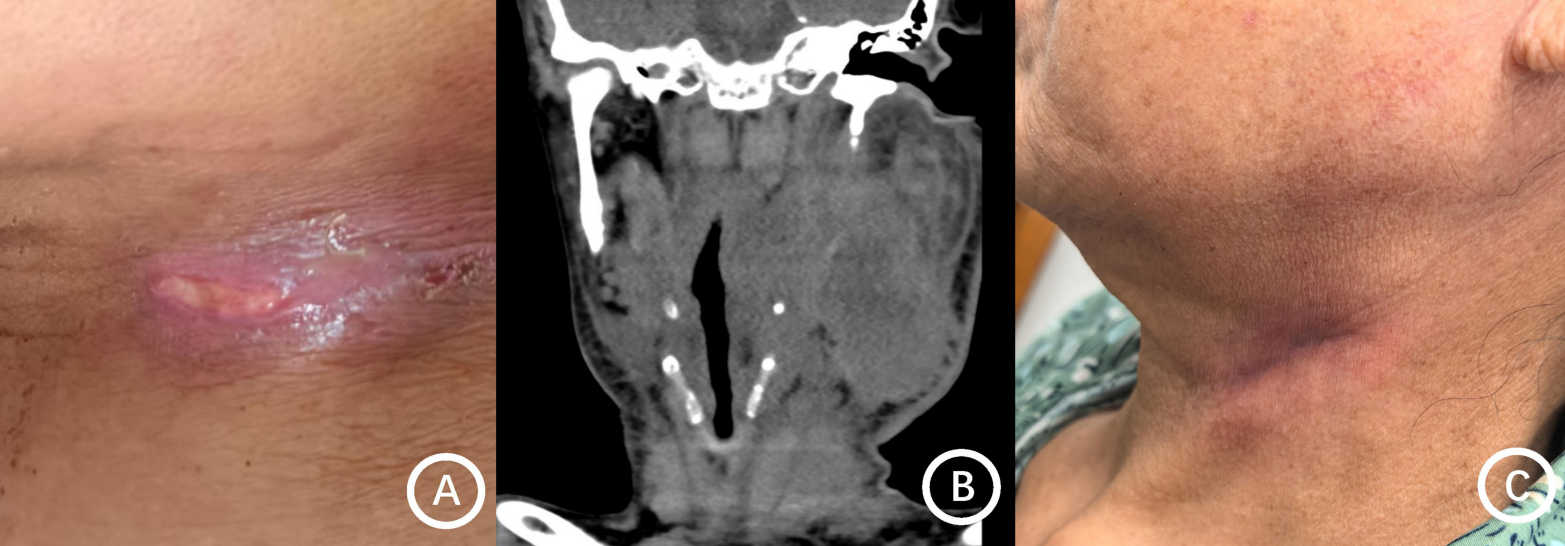
Clinical and imaging findings of the neck in patient 7. **(A)** Erythema, swelling, ulceration, and purulent drainage are visible on the left side of the neck. **(B)** Axial neck CT scan shows a conglomerate, mass-like soft tissue density lesion in left levels II and III. **(C)** Appearance of the neck following treatment.

**Figure 3:**
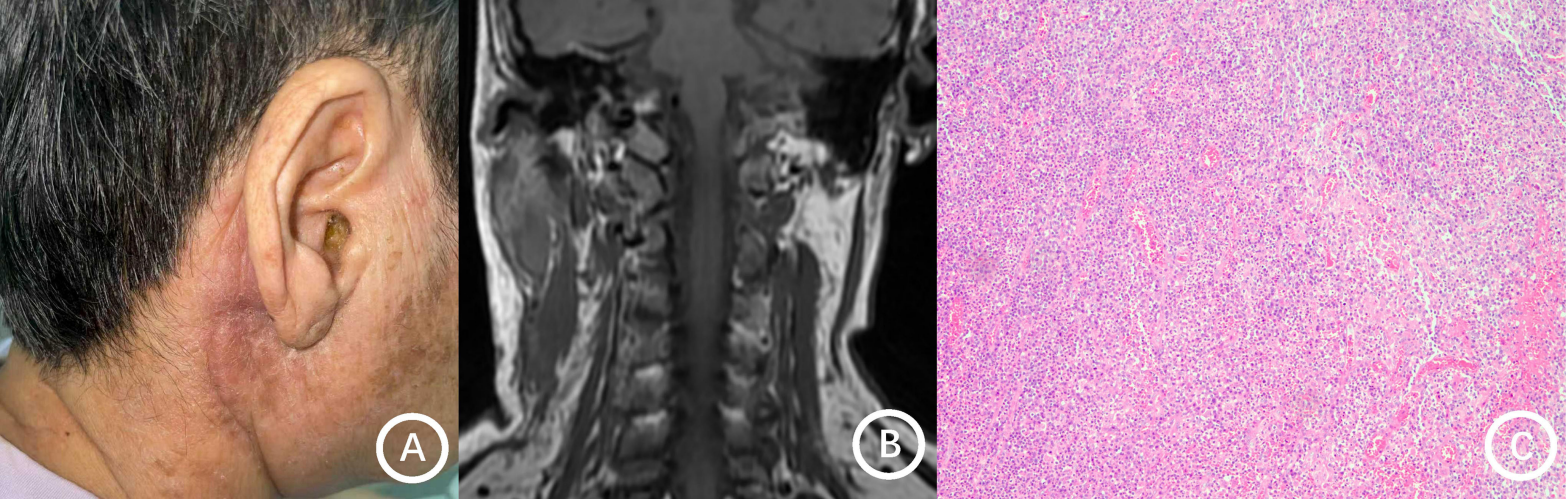
Clinical and imaging findings of the neck in patient 13. **(A)** Diffuse swelling of the right neck without overlying erythema or ulceration. **(B)** Neck MRI shows multiple enlarged lymph nodes in the right retropharyngeal lateral group, within the parotid gland, and on both sides of the neck. **(C)** Histopathological examination (hematoxylin and eosin staining; original magnification, 10x) reveals an infiltrate of plasma cells and neutrophils, findings consistent with chronic suppurative inflammation.

The median time from symptom onset to diagnosis was 5 months (range, 1–19 months). The most common presenting symptoms were neck swelling and pain, although some patients presented with asymptomatic neck masses (**Figures 2**, **3**). Misdiagnosis as tuberculosis, malignancy, or lymphoma was common. Among the 13 patients, nine were diagnosed with NTM infection, including *M. abscessus* (3 cases), *M. fortuitum* (2 cases), *M. gordonae* (2 cases), and one case each of *M. colombiense* and *M. intracellulare*. The remaining four cases were infected with TM. NGS was the most frequently used diagnostic method for NTM (8 cases), whereas culture was the primary method for TM (4 cases) (**Table 3**).

**Table 3:** Neck mass findings.

ID	Symptoms	Misdiagnosed diseases	pathogenic bacteria	Diagnostic methods	Histophathological results	Onset-to-diagnosis time	Therapy	Clinical outcome	Follow-up duration (months)/ number of exacerbations	Clinical endpoint
P1	None	TB	*M. fortuitum*	Histopathology	Granulomatous inflammation	18 months	CLR, MXF, LNZ, IPM	Improved	63/7	Alive
P2	Redness, swelling, pain	TB	*M. abscessus*	NGS	Chronic granulomatous inflammation	9 months	AZM, AMK, MXF, CFX	Improved	33/0	Alive
P3	Swelling, pain	Cancer	*M. abscessus*	NGS	Inflammatory changes	1 month	AZM, AMK, MXF, CFX	Improved	20/2	Alive
P4	Ulceration with purulent discharge, pain	Lymphadenitis	*M. gordonae*	NGS	Chronic granulomatous inflammation	4 months	AZM, EMB, AMK, MXF, RIF, CFX, LNZ, IPM	Improved	32/0	Alive
P5	None	Cancer	*M. intracellulare*	NGS	Chronic granulomatous inflammation	5 months	EMB, MXF, RIF, LNZ	Improved	28/0	Alive
P6	None	Cancer	*M. colombiense*	NGS	Chronic inflammation	2 months	AZM, AMK, LNZ, IPM	Death	40/2	Death
P7	Redness, swelling, pain	Lymphoma	TM	NGS, culture	ND	1 month	ITCZ	Improved	1/1	Alive
P8	None	TB	*M. gordonae*	NGS	Chronic granulomatous inflammation	12 months	AZM, EMB, MXF, RIF, CFX, LNZ, IPM	Improved	45/2	Alive
P9	None	Cancer	TM	Culture	Purulent inflammation	5 months	AMB, VRCZ, ITCZ	Improved	53/0	Alive
P10	Redness, swelling, pain	TB	TM	Culture	Purulent inflammation	19 months	AMB, VRCZ, ITCZ	Improved	18/0	Alive
P11	None	TB	TM	Culture	Chronic granulomatous inflammation	5 months	AMB, VRCZ, ITCZ	Improved	86/0	Alive
P12	Pain	Cancer	*M. fortuitum*	NGS	Granulomatous inflammation	10 months	CLR, AZM, MXF, IPM	Improved	19/1	Alive
P13	Pain	Lymphadenitis	*M. abscessus*	NGS	Chronic purulent inflammation	6 months	AZM, AMK, CFX	Improved	1/1	Alive

CLR, Clarithromycin; MXF, Moxifloxacin; LNZ, Linezolid; IPM, Imipenem; AZM, Azithromycin; AMK, Amikacin; CFX, Cefoxitin; EMB, Ethambutol; RIF, Rifampicin; AMB, AmphotericinB; VRCZ, Voriconazole; ITCZ, Itraconazole.

Histopathological examination predominantly revealed chronic granulomatous inflammation (7 cases), chronic inflammation (2 cases), purulent inflammation (2 cases), granulomatous inflammation (1 case), and inflammatory changes (1 case) (**Figure 3**; **Table 3**).

### Clinical outcomes and follow-up

All 13 patients received pathogen-directed antimicrobial therapy, and six underwent surgical incision and drainage. Among them, 12 achieved clinical improvement or infection control following treatment, while one patient died. The median follow-up duration was 28 months (range: 1–86 months). During the follow-up period, disease exacerbations occurred in 6 patients (46.2%), with a total of 15 exacerbation events recorded (median: 2 events per patient; range: 1–7). Among the 12 patients who achieved clinical improvement, all remained stable at the last follow-up, with no further disease progression (**Table 3**).

## Discussion

Neck masses, as a common clinical manifestation, have complex etiologies that may include infectious, neoplastic, and immune-related diseases, among other possibilities (Liang et al., 2026). This study reviewed 13 cases of neck masses as the initial presentation of TM or NTM infections. Both pathogens can manifest as chronic cervical lymphadenopathy, often with low-grade fever and weight loss, and are easily misdiagnosed as tuberculosis, lymphoma, or metastatic cancer. Histopathologically, TM infection typically presents as suppurative inflammation, whereas NTM infection often shows non-specific granulomatous inflammation. Microabscesses, ill-defined granulomas, non-caseating granulomas, and few giant cells are characteristic of NTM lymphadenitis (Reid and Wolinsky, 1969; Kraus et al., 1999). However, early-stage tuberculous lymphadenitis may also lack caseation, and histopathological features overlap with NTM, making differentiation challenging. TM typically induces granulomatous, suppurative, or reactive necrotizing inflammation (Yang et al., 2022). Therefore, pathological findings for TM and NTM lymph node involvement are non-specific. Patients often initially present to otolaryngology departments, where tuberculosis or malignancy is suspected, leading to surgical excision and histopathological examination while pathogen screening is overlooked. Thus, mNGS and culture are essential for detecting TM and NTM, particularly when conventional anti-tuberculosis or antibacterial therapy fails.

The 13 patients included in this study were all HIV-negative and long-term residents of the Guangxi region. Traditionally, TM infection has been considered more common in HIV/AIDS patients; however, in recent years, there have been increasing reports of TM co-occurring with immunodeficiency associated with AIGAs, autoimmune diseases, the use of corticosteroids and/or immunosuppressants, as well as in patients with malignant tumors (Qiu et al., 2021). Pediatric NTM cervical lymphadenitis typically occurs in immunocompetent children, whereas adult cases warrant evaluation for IFN-γ/IL-12 pathway defects or immunosuppression (Ramirez-Alejo and Santos-Argumedo, 2014). Given the high prevalence of AIGAs in Guangxi, all patients underwent AIGAs testing and tested positive. Immunodeficiency caused by AIGAs was first described in 2004 as a novel syndrome of adult-onset immunodeficiency (Döffinger et al., 2004; Chan et al., 2016). IFN-γ plays a crucial role in the body’s defense against intracellular pathogens. Elevated antibody titers can inhibit IFN-γ signal transduction as well as the production of TNF-α and IL-12, leading to severe impairment of the Th1 response. This compromises the body’s ability to combat pathogens, particularly intracellular infectious agents such as TM, NTM, *non-typhoidal Salmonella*, *Burkholderia cepacia*, *varicella-zoster virus*, and *cytomegalovirus* (Browne et al., 2012; Chi et al., 2016). Thus, AIGAs likely underlie the susceptibility to TM/NTM infections in this cohort.

In addition to neck involvement, all patients had disseminated infections. Currently, there is no standard treatment protocol for TM infections in HIV-negative hosts. Clinical guidelines for treatment primarily refer to the management of TM-infected patients who are HIV-positive, which involves intravenous amphotericin B followed by oral itraconazole as maintenance therapy (Kaplan et al., 2009; Wang et al., 2023). In this cohort, antifungal treatment involving amphotericin B, itraconazole, and voriconazole yielded relatively satisfactory clinical outcomes. The current approach to treating NTM infection involves the use of at least three antibiotics. Most regimens include ethambutol and rifampin, with a macrolide backbone such as clarithromycin or azithromycin (Griffith et al., 2007). For pediatric NTM cervical lymphadenitis, surgical intervention is the preferred treatment and is considered superior to antibiotic therapy (Lindeboom et al., 2007). However, in our adult patients, surgical intervention alone often resulted in poor wound healing or recurrence, whereas pathogen-directed antimicrobial therapy led to significant improvement. Evidence comparing surgical versus antibiotic therapy for adult cervical TM or NTM lymphadenitis is limited, and the small sample size of this study precluded a comparative analysis.

Prevention of infections caused by special pathogens such as TM and NTM focuses on reducing environmental exposure and strengthening protection for high-risk populations. In endemic areas, immunocompromised individuals (e.g., those with AIDS or malignant tumors) should avoid entering high-risk environments such as bamboo forests, and refrain from contacting or consuming animal hosts like bamboo rats (Gugnani et al., 2004). Systematic surveillance for TM infections should be implemented, including among non-HIV patients and in non-traditional endemic regions (Fang et al., 2026; Miao et al., 2026). Furthermore, strengthening water source management is essential for the prevention and control of nontuberculous mycobacteria, and enhancing early recognition and healthcare-seeking awareness is also critical (Norton et al., 2020).

Unlike previous studies that primarily focused on disseminated infections associated with AIGAs syndrome, the present study specifically characterizes TM/NTM infections presenting with neck masses as the initial manifestation—a clinically important but underrecognized disease entity. Previous cohort studies have shown that patients with higher AIGAs titers (≥1:2500) exhibit more severe disease involving multiple organ systems, along with prolonged diagnostic delays, suboptimal treatment responses, and an increased requirement for glucocorticoid therapy to modulate immune function (Liang et al., 2024). Based on these findings, we propose the following recommendations for patients presenting with neck masses: (1) histopathological examination should be complemented by microbial culture, particularly when purulent discharge is present, with attention to specific pathogens beyond common infectious agents; (2) AIGAs testing is recommended for HIV-negative patients with TM/NTM infections, as titer levels may help guide treatment strategies; (3) early empiric broad-spectrum antimicrobial therapy covering TM, NTM, and bacteria should be initiated, with more aggressive intervention warranted in patients with high AIGAs titers.

## Data Availability

The datasets presented in this study can be found in online repositories. The names of the repository/repositories and accession number(s) can be found in the article/supplementary material.
